# The Diseases and Casualties for the Year 1800

**Published:** 1801-03

**Authors:** 


### The DISEASES and CASUALTIES for the YEAR 1800.

| | | | | | |
|---|---|---|---|---|---|
| Abortive and Stillborn | 492 | Gravel, Stone, and Stranguary | 16 | Surfeit | 2 |
| Abfcefs | 37 | Grief | 5 | Swine Pox | 1 |
| Aged | 1742 | Headmouldfhot, Horfefhoehead, and Water in the head | 80 | Teeth | 414 |
| Ague | 2 | Head-ach | 2 | Thrufh | 33 |
| Apoplexy & Suddenly | 252 | Jaundice | 67 | Vomiting and Loofenefs | 2 |
| Afthma and Phthific | 801 | Jaw Locked | 1 | Worms | 15 |
| Bedridden | 3 | Inflammation | 593 | Broken Limbs | 9 |
| Bleeding | 9 | Leprofy | 1 | Bruifed | 4 |
| Burften and Rupture | 15 | Lethargy | 2 | Burnt | 12 |
| Cancer | 58 | Livergrown | 3 | Drowned | 124 |
| Chicken Pox | 1 | Lunatick | 162 | Exceffive Drinking | 12 |
| Childbed | 164 | Lumbago | 1 | Executed * | 19 |
| Colds | 27 | Meafles | 395 | Found Dead | 10 |
| Colick, Gripes, and Twifting of the Guts | 11 | Mifcarriage | 4 | Fractured | 1 |
| Confumption | 5721 | Mortification | 242 | Frighted | 2 |
| Convulfions | 4512 | Palpitation of the Heart | 2 | Frozen | 2 |
| Cough, and Hooping Cough | 380 | Palfy | 122 | Killed by Falls and other Accidents | 62 |
| Cow Pox | 1 | Pleurify | 37 | Killed by Fighting | 3 |
| Cramp | 3 | Quinfy | 1 | Killed themfelves | 29 |
| Croup | 13 | Rafh | 1 | Murdered | 4 |
| Dropfy | 1003 | Rheumatifm | 4 | Poifoned | 2 |
| Eaten by Lice | 1 | Scurvy | 5 | Scalded | 6 |
| Evil | 6 | Small Pox | 2409 | Shot | 1 |
| Fevers of all kinds | 2712 | Sore Throat | 1 | Smothered | 1 |
| Fiftula | 6 | Sores and Ulcers | 8 | Starved | 8 |
| Flux | 9 | Spafm | 2 | Suffocated | 3 |
| French Pox | 27 | Stoppage in the Stom. | 8 | | |
| Gout | 105 | | | Total | 314 |

Increafed in the Burials this Year 4934.

* *There have been executed in Middlesex and Surry* 27; *of which number (*19 *only) have been reported to be buried (as such) within the Bills of Mortality.*

Chriftened

| | | | |
|---|---|---|---|
| Christened | Males - 10112 | Females - 9064 | In all 19176 |
| Buried | Males - 11605 | Females - 11463 | In all 23608 |

Whereof have died,

| | | | |
|---|---|---|---|
| Under Two Years of Age - - | 6657 | Seventy and Eighty - - - - | 1459 |
| Between Two and Five - - - | 2553 | Eighty and Ninety - - - - | 655 |
| Five and Ten - - - - - - | 848 | Ninety and a Hundred - - - | 97 |
| Ten and Twenty - - - - - | 710 | A Hundred and One - - - - | 1 |
| Twenty and Thirty - - - - | 1582 | A Hundred and Four - - - | 2 |
| Thirty and Forty - - - - - - | 2055 | A Hundred and Six - - - - | 1 |
| Forty and Fifty - - - - - | 2308 | A Hundred and Seven - - - | 1 |
| Fifty and Sixty - - - - - | 2163 | A Hundred and Eighteen - - | 1 |
| Sixty and Seventy - - - - | 1973 | A Hundred and Twenty - - - | 2 |

